# Prediction of ESRD in Pauci-immune Necrotizing Glomerulonephritis: Quantitative Histomorphometric Assessment and Serum Creatinine

**DOI:** 10.1053/j.ajkd.2009.10.047

**Published:** 2010-02

**Authors:** Clara J. Day, Alec J. Howie, Peter Nightingale, Shazia Shabir, Dwomoa Adu, Caroline O. Savage, Peter Hewins

**Affiliations:** 1 Department of Nephrology, University Hospitals Birmingham NHS Foundation Trust, Birmingham, UK; 2 Department of Pathology, University College London, UK; 3 Wellcome Trust Clinical Research Facility, University Hospitals Birmingham NHS Foundation Trust, Birmingham, UK; 4 School of Immunity & Infection, College of Medical and Dental Sciences, University of Birmingham, Birmingham, UK

**Keywords:** Vasculitis, necrotizing glomerulonephritis, antineutrophil cytoplasm autoantibody, kidney biopsy, end-stage renal disease (ESRD)

## Abstract

**Background:**

Clinical and pathologic features that predict outcome have important potential application in patients with pauci-immune necrotizing glomerulonephritis (usually antineutrophil cytoplasmic antibody–associated vasculitis). This study examines the predictive value of simple quantitative renal histologic measurements in a large cohort with extended follow-up.

**Study Design:**

Cohort study.

**Setting & Participants:**

390 consecutive patients with pauci-immune necrotizing glomerulonephritis at a single hospital (1983-2002); 90 patients underwent repeated kidney biopsy during follow-up.

**Predictors:**

Age and serum creatinine concentration at biopsy, antineutrophil cytoplasmic antibody specificity, percentage of normal glomeruli, percentage of glomeruli with active lesions, and index of chronic damage (quantitative measurement of established cortical damage) in the initial kidney biopsy for all patients. The same factors were assessed in both biopsy specimens for patients undergoing an additional biopsy.

**Outcomes & Measurements:**

End-stage renal disease and patient survival.

**Results:**

Mortality at 1 and 5 years was 23% and 40%, respectively: standardized mortality ratio, 4.74 (95% CI, 3.62-6.32). End-stage renal disease was reached by 14% and 18% at 1 and 5 years, respectively. In multivariable analysis, serum creatinine level at biopsy and percentage of normal glomeruli in the initial biopsy specimen were the best predictors of kidney survival. C Statistics were 0.80 for creatinine level alone and 0.83 for creatinine level with normal glomeruli. In patients undergoing an additional biopsy, rapid progression in the index of chronic damage and serum creatinine level at the second biopsy were associated with kidney survival in multivariable analysis.

**Limitations:**

Retrospective analysis. External validity of the index of chronic damage requires further assessment. Selection bias may influence repeated biopsy analyses.

**Conclusions:**

Serum creatinine level at biopsy best predicts kidney survival in patients with pauci-immune necrotizing glomerulonephritis overall.

Wegener granulomatosis and microscopic polyangiitis are antineutrophil cytoplasmic antibody (ANCA)-associated small-vessel vasculitides that cause rapidly progressive pauci-immune necrotizing glomerulonephritis.^1^ The routine use of immunosuppressive therapies has dramatically improved prognosis,^2,3^ and randomized trials have established evidence-based treatment.^4,5^ Nevertheless, significant morbidity and mortality are still prevalent as a result of both disease and treatment, particularly in the elderly.^6^

Previous reports have correlated presenting serum creatinine level with kidney outcome in patients with pauci-immune necrotizing glomerulonephritis;^7,8^ however, at least some patients with severe kidney disease recover independent kidney function when treated appropriately.^5^ Whereas the primary purpose of a kidney biopsy is diagnostic, it not infrequently reveals a combination of acute and chronic lesions that may have prognostic relevance. A number of studies have highlighted the value of measurements of glomerular morphologic characteristics in this context; however, assessments of tubular and interstitial damage frequently are semiquantitative and may be subject to greater variability. Moreover, assessments that involve many components are not necessarily readily applicable in routine practice outside of specialist centers.^7,9^

We have previously described a simple morphometric measure of chronic damage that assesses the entire area of renal cortex in a biopsy specimen and have shown that the index predicts kidney survival in various settings.^10-12^ We hypothesized that the index would predict kidney survival in patients with pauci-immune necrotizing glomerulonephritis and, in this study, set out to test its performance in comparison to quantitative measurements of glomerular morphologic characteristics in a retrospective cohort of 390 patients sampled at a single center.

## Methods

### Clinical Data

All patients at the Queen Elizabeth Hospital, Birmingham, UK, with a diagnosis of pauci-immune necrotizing glomerulonephritis by kidney biopsy between 1983 and 2002 were included. Clinical data were obtained from patient records (hospital and general practice). Date and cause of death were obtained from public records and death certificates. Serum creatinine was measured at the time of biopsy or before the initiation of acute dialysis therapy if this occurred before the biopsy was performed. Biopsies ordinarily were performed before administration of immunosuppressive therapy or shortly thereafter in exceptional cases. Repeated biopsies ordinarily were performed before escalation of immunosuppression. Patients were defined as reaching the end point of end-stage renal disease (ESRD) when they required permanent renal replacement therapy, in other words, long-term dialysis therapy or transplant. Patients who required acute dialysis at presentation or relapse, but who recovered independent kidney function after treatment, were not classified as reaching the end point. Time to the end point was defined as time elapsed between biopsy (time zero) and either death or initiation of permanent renal replacement therapy. The status of all patients was determined at the end of 2004. Research was conducted in accordance with the Declaration of Helsinki.

During the period studied, standard treatments used were cyclophosphamide (either pulsed intravenous or daily oral) for active disease with high-dose prednisolone (1 mg/kg) tapered over 2-3 months. On remission and after 3 to 6 months of treatment, patients were switched to maintenance therapy with azathioprine and low-dose prednisolone. Few patients stopped the medication. Those with either dialysis-requiring acute kidney failure or pulmonary hemorrhage were treated using plasma exchange.

Death certificates were available for 186 of 214 patients who died. Forty-three patients died within 3 months of diagnosis, and certified causes of death in these cases were kidney failure, vasculitis, or infection. We classified all deaths during this period as related to active vasculitis because infection was presumed to be secondary to intensive immunosuppression. Deaths attributed to infection > 3 months after diagnosis were coded separately.

### Measurement of Index of Chronic Damage and Other Pathologic Investigations

Stored slides of renal biopsy specimens were examined retrospectively without knowledge of patient details and irrespective of the number of glomeruli in the specimen. On a representative section, usually stained using periodic acid–methenamine silver, the total number of glomeruli was counted. Each glomerulus was put into 1 of 5 categories, which were summed for each biopsy and expressed as percentages of the total number of glomeruli: normal glomeruli, globally sclerosed glomeruli, glomeruli with ischemic shrinkage, glomeruli with active vasculitic lesions, and glomeruli with healed vasculitic lesions. Active vasculitic lesions were defined as segmental or global areas of combinations of thrombosis, disruption of capillary basement membranes, and cells in Bowman space. Healed vasculitic lesions were defined as those in which areas of fibrosis included the glomerular tuft and Bowman space. Active and healed segmental lesions usually were sharply demarcated from the rest of the glomerulus. Lesions that were healing were arbitrarily assigned to active or healed categories based on the relative amounts of cells and fibers in the lesions. Arteritis or arteriolitis was diagnosed by the finding of fibrinoid necrosis and/or an inflammatory infiltrate in the wall of relevant vessels.

The index of chronic damage was measured in each biopsy specimen as previously described.^10-12^ Briefly, using a digital image of renal cortex, atrophic areas were outlined by freehand and their cross-sectional areas were measured in pixels. Atrophic areas were defined as those containing any combination of globally sclerosed glomeruli, shrunken tubules with thickened basement membranes, interstitial fibrosis, cysts, and occluded vessels. The cortex in the image then was outlined and its area was measured. This was repeated until all cortex was measured. The total of atrophic areas was expressed as a percentage of total cortical area and called the index of chronic damage. This could range from 0% in a kidney without no chronic damage to 100% in a completely atrophic kidney.

Previously, the limits of interobserver agreement for the index of chronic damage were determined to be between 0.88 (95% confidence interval [CI], 0.82-0.93) and 1.22 (95% CI, 1.15-1.30), with a mean agreement of 1.03 (95% CI, 0.99-1.07).^11^ The limits of intraobserver agreement were 0.79 (95% CI, 0.74-0.85) and 1.29 (95% CI, 1.20-1.39), with a mean of 1.01 (95% CI, 0.97-1.06).^11^ The definition of the index of chronic damage is listed in Box 1.

### Data Analysis

Analysis was performed using SPSS for Windows, version 14 (SPSS Inc, www.spss.com/uk/). Univariable and multivariable survival analyses were performed initially using stepwise Cox regression with creatinine level as a continuous variable. Histologic parameters were divided into groups of 1 standard deviation to make hazard ratios more meaningful. When > 1 biopsy was performed, only the initial presenting biopsy specimen was used in these analyses. Time origin (t = 0) was the value of the parameter at the initial presenting biopsy. If a patient did not progress to ESRD, they were censored at either death or last follow-up, with the latter at least 2 years after the initial presentation. Correlations of pathologic variables with serum creatinine concentrations at biopsy or ANCA status were computed using Spearman rank correlation for nonparametric variables. The C statistic is the proportion of pairs of individuals for which the model correctly predicted which individual had the shorter time to event (pairs for which this is not known were excluded from the calculation).

In analysis of the repeated biopsy data, when there were > 2 biopsies available, only the earliest and most recent were analyzed. Patients underwent a repeated biopsy because of either a suspected disease flare or inadequate response to therapy. As such, this group is subject to selection bias. Rates of changes in the index of chronic damage, percentage of normal glomeruli, and serum creatinine concentration were calculated by dividing the change in each parameter between the 2 biopsies by the time separating the 2 samples.

Further analysis of the initial biopsy data was performed by dividing the population into quartiles of percentage of normal glomeruli. Quartile division was not possible for the index of chronic damage because a large number of biopsy specimens showed an index of 0%. Therefore, this parameter was divided into 4 groups: all biopsy specimens with an index of 0% and the remainder of the population divided into tertiles. Differences between groups in progression to ESRD were calculated using stepwise Cox regression. A likelihood ratio comparing goodness of fit was used to test for interactions between creatinine level and 2 histologic variables (percentage of normal glomeruli and index of chronic damage) in multivariable analysis of the entire population.

## Results

### Baseline Characteristics and Overall Outcomes

During the study period, 390 patients were given a diagnosis of pauci-immune necrotizing glomerulonephritis. Of these, 90 patients underwent > 1 kidney biopsy, but only initial biopsies were included in the analysis unless otherwise stated (see Repeated Biopsy Data in this section). Median age at initial biopsy was 64 years (range, 15-90 years), and median serum creatinine level at biopsy was 3.86 mg/dL (range, 0.52-20.6 mg/dL). Follow-up was until patient death or for at least 2 years from the initial biopsy for surviving patients, with median follow-up of 166 weeks (range, 1 day-1,256 weeks). During follow-up, 214 (55%) patients died: 89 (23%) during the first year, 149 (40%) by 5 years, and 184 (49%) by 10 years. Permanent renal replacement therapy was required in 87 (22%) patients: 54 (14%) in the first year, 67 (18%) by 5 years, and 76 (21%) by 10 years.

In the present cohort, the median index of chronic damage was 10% (range, 0%-94%). Baseline characteristics and outcomes for patients segregated by index of chronic damage values are listed in Table 1. Median percentage of normal glomeruli was 31% (range, 0%-100%), and median percentage of glomeruli with active lesions was 19% (range, 0%-100%). ANCA immunofluorescence data were available for 230 patients. Perinuclear ANCA (pANCA)-positive patients were significantly older at presentation than cytoplasmic ANCA (cANCA) patients (median age, 67 vs 57 years; *P* < 0.001). The mean index of chronic damage was significantly higher in pANCA-positive patients (31.17% vs 19.55%; *P* = 0.003), which remained significant after correction for age (*P* = 0.04). Furthermore, pANCA-positive patients had a higher percentage of globally sclerosed glomeruli (*P* < 0.001), whereas cANCA-positive patients showed more glomeruli with active lesions (*P* = 0.001) and arteritis (*P* = 0.03).

### Mortality

Death was predicted by serum creatinine level at biopsy (hazard ratio, 1.12; 95% CI, 1.09-1.16; *P* < 0.001) and age (hazard ratio, 1.05; 95% CI, 1.04-1.07; *P* < 0.001), but not by any histologic variable examined. An association between pANCA versus cANCA positivity and increased mortality (*P* = 0.03) was not significant after correction for age.

Only 2 patients died of active vasculitis > 3 months after diagnosis. During the entire study period, major causes of death were as follows: active vasculitis or complications of therapy within 3 months of diagnosis, 45 (24%); ischemic heart disease or stroke, 42 (23%); infection > 3 months from diagnosis, 41 (22%); chronic kidney disease, 32 (17%); malignancy 20 (11%); and gastrointestinal disease, 6 (3%).

Using data obtained from the UK Office of National Statistics for age-standardized death rates per 1,000 population in 2002, the calculated standardized mortality ratio for this patient cohort was 4.74 (95% CI, 3.62-6.32) with an odds ratio of 9.32 (95% CI, 6.45-13.47).

### Correlation of Variables With Serum Creatinine Concentration at Biopsy

There were significant correlations between serum creatinine level at biopsy and age (*r* = 0.273; *P* < 0.001), index of chronic damage (*r* = 0.263; *P* < 0.001), percentage of normal glomeruli (*r* = −0.583; *P* < 0.001), percentage of glomeruli with active lesions (*r* = 0.368; *P* < 0.001), and percentage of globally sclerosed glomeruli (*r* = 0.192; *P* < 0.001). There was no correlation between serum creatinine level at biopsy and either ANCA subtype (*r* = 0.033; *P* = 0.6) or presence of arteritis (*r* = −0.51; *P* = 0.4).

### Progression to ESRD

Using univariable analysis, serum creatinine level at biopsy and all histologic variables examined significantly correlated with ESRD both at 1 year and throughout the duration of the study. In contrast, age was not associated with ESRD (Table 2). In multivariable analysis, serum creatinine level at biopsy and percentage of normal glomeruli were independent predictors of ESRD (Table 3). This was reflected in C statistics for Cox regression: C = 0.8 for creatinine level alone and C = 0.83 for creatinine level with percentage of normal glomeruli. No significant interaction was shown between serum creatinine level and either percentage of normal glomeruli or index of chronic damage. Considering those for whom ANCA immunofluorescence was known, there was no difference in progression to ESRD between pANCA- and cANCA-positive patients (*P* = 0.9).

Dividing the index of chronic damage into groups suggested a threshold effect because kidney outcome did not differ significantly for the first 3 groups. However, the fourth group (≥48% chronic damage) had a significantly worse kidney outcome (Table 1). A threshold effect also was apparent for normal glomeruli because progression to ESRD did not differ significantly for the third (32%-65%) compared with the fourth quartile (≥66%), whereas progression to ESRD was significantly more frequent for the first (0%-8%) and second (9%-31%) quartiles (ie, biopsy specimens showing the least normal glomeruli; Table 4). Conversely, the second, third, and fourth quartiles of normal glomeruli had better kidney outcomes than the first quartile (≤8%; not shown).

### Repeated Biopsy Data

**Figure 1:**
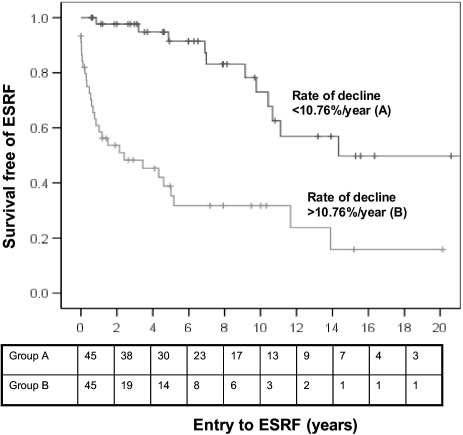
Progression to end-stage renal failure (ESRF) stratified by rate of decrease in index of chronic damage between the first and last renal biopsy specimens (*P* < 0.001). Values below the plot indicate numbers remaining in the analysis at each time.

Data confirm that histologic variables measured in a first biopsy specimen are associated with ESRD across the entire patient cohort. In this study, 90 patients underwent > 1 biopsy, providing an opportunity to examine the association of changes in histologic variables with outcomes of individual patients. Median time between biopsies was 397 days (range, 9-6,666 days). Comparing patients in the subgroup that developed ESRD (n = 40) with those who did not (n = 50), second biopsy specimens from those reaching ESRD showed a higher median index of chronic damage (66.5% vs 29.0%) and decreased median percentage of normal glomeruli (6.0% vs 20.0%). In multivariable analysis, only creatinine level at the second biopsy (median, 7.52 mg/dL in patients developing ESRD vs 2.59 mg/dL in those retaining independent kidney function) and rate of change in index of chronic damage (0.11%/d vs 0.009%/d) were associated with progression to ESRD (Table 5). For the entire subgroup, median rate of change in the index of chronic damage was +10.76%/y (Fig 1).

## Discussion

The prognosis for patients with pauci-immune necrotizing glomerulonephritis has improved with therapeutic immunosuppression.^2,3^ Nonetheless, by 5 years, 18% of patients in this cohort required permanent renal replacement therapy and 40% had died. Previous studies also have reported high mortality in patients with systemic vasculitis.^21,23^ Allowing for the limitations of accuracy in death certification, the high rate of infection-related mortality in this study supports the hypothesis that adverse effects of therapy contribute to poor outcome and emphasizes the advantage of identifying patients with salvageable kidney function.

Previous smaller studies have reported more long-term damage in pANCA/myeloperoxidase-ANCA–positive versus cANCA/proteinase 3–ANCA patients, but similar outcomes.^9,19-22^ Our study supports this concept and indicates that ANCA subtype should not influence treatment. The basis of the difference in pathologic features between the 2 subgroups is uncertain and could reflect either earlier diagnosis in patients with cANCA (perhaps because of prominent extrarenal symptoms) or genuinely divergent disease characteristics.

Other studies of kidney biopsies in patients with systemic vasculitis (Table 6) have involved relatively complex scoring systems.^7,9,17,22,25,26^ Moreover, the consistency of biopsy interpretation in patients with pauci-immune necrotizing glomerulonephritis is uncertain because although quantitative measurements of glomerular morphologic characteristics show satisfactory agreement, interobserver agreement for dichotomous variables used to score tubulointerstitial damage appears to be poor.^13^ Similarly, the reproducibility and predictive value of semiquantitative chronicity scores in patients with lupus nephritis is controversial, and their applicability outside specialist referral centers is disputed.^14,15^ More recently, computer-assisted morphometric assessment of lupus nephritis has been described and has predicted outcome in a relatively small cohort (n = 48).^16^

Our study comprises a large cohort with a unified diagnosis and extended follow-up. Serum creatinine level at biopsy correlated with glomerular morphologic characteristics, as previously reported,^7,8^ and with index of chronic damage. Furthermore, both glomerular morphologic characteristics and the index of chronic damage significantly correlated with kidney outcome. Multivariable analysis showed serum creatinine level at biopsy and percentage of normal glomeruli in the initial biopsy specimen to be independent predictors of ESRD. As in other studies, no pathologic features predicted death, which was predicted by age and serum creatinine level at biopsy.

In a subgroup of patients undergoing repeated biopsy (performed in the context of suspected disease flare or inadequate response to therapy), accelerated change in the index of chronic damage was associated independently with kidney outcome, whereas percentage of normal glomeruli was not. Previous studies using repeated biopsy data have shown progression of chronic damage (semiquantitatively scored)^17^ or an increase in global glomerulosclerosis,^18^ but not an association with progression to ESRD. The index of chronic damage might provide additional information because it reflects irreversible damage in the tubulo-interstitial and vascular compartments, as well as glomerular scarring. Furthermore, the index may be more meaningful when a biopsy specimen contains only a few glomeruli. We have already reported that the index is an indicator of progression to ESRD in patients with lupus nephritis^11^ and in a heterogeneous cohort of patients with chronic kidney disease^10^ (predominantly immunoglobulin A nephropathy and Henoch-Schönlein nephritis).

There are several limitations to our study. It was a retrospective analysis with uncontrolled differences between patient groups, mainly in treatment regimens. ANCA specificity was analyzed using indirect immunofluorescence rather than enzyme-linked immunosorbent assay and was recorded in only 59% (230/390) of patients, reflecting the available data set, rather than an unusually high proportion of truly ANCA-negative cases. In addition, there has been only limited assessment of the extent of inter- and intraobserver agreement using the index of chronic damage. Previous studies involving a small number of pathologists have shown that the degree of variation is low,^10^ but this merits confirmation. Furthermore, repeated biopsy analyses could be affected by selection bias.

Although histologic variables can correlate with or predict kidney outcome, histology has not yet been used to direct treatment in a trial setting. An analysis of biopsies from the MEPEX (methyl prednisolone or plasma exchange for severe renal vasculitis) trial concluded that for patients receiving plasma exchange, the chance of kidney recovery always exceeds the risk of therapy-related death regardless of the severity or nature of histopathologic change.^24^ This suggests that treatment should not be restricted for patients who present with severely decreased kidney function. Nevertheless, the likelihood of reaching ESRD or dying is substantial using any current treatment, and death from complications of therapy is well recognized. Accordingly, the search for robust tools that could support treatment decisions (either limitation when it is futile or intensification when the risk of inadequate response is greater) remains relevant.

## Figures and Tables

**Table 1:** Index of Chronic Damage, Baseline Characteristics, and Outcomes

Group	Index of Chronic Damage (%)	Age (y)	SCr at Presentation (mg/dL)	Progression to ESRD				Death			
				% Reaching Outcome	Time to Outcome (d)	*P*	HR (95% CI)	% Reaching Outcome	Time to Outcome (d)	*P*	HR (95% CI)
1 (n = 108)	0	56 (15-86)	2.35 (0.52-18.05)	19.1	110 (0-4,052)	—	1.00 (reference)	46.0	319 (2-8,309)	—	1.00 (reference)
2 (n = 88)	5 (1-11)	64 (26-85)	3.55 (0.79-13.72)	18.1	201 (0-5,071)	0.5	0.78 (0.39-1.51)	52.2	637 (9-5,099)	0.6	0.9 (0.60-1.36)
3 (n = 88)	23 (11-49)	65 (18-90)	3.35 (0.89-17.15)	20.4	245 (0-6,224)	0.9	1.05 (0.55-2.00)	62.5	1,269 (12-7,519)	0.2	1.31 (0.89-1.94)
4 (n = 86)	73.5 (50-94)	68 (26-86)	5.78 (1.17-20.64)	34.9	14 (0-5,234)	0.002	2.42 (1.36-4.33)	68.1	529 (8-5,096)	0.004	1.75 (1.19-2.58)

*Note:* Values expressed as median (range). Groups are all patients with no chronic damage on biopsy (index of chronic damage, 0%; group 1) and the remainder of the population divided into tertiles of index of chronic damage (groups 2, 3, and 4). Global *P* = 0.001 for progression to ESRD and *P* = 0.003 for death. Abbreviations: CI, confidence interval; ESRD, end-stage renal disease; HR, hazard ratio; SCr, serum creatinine.

**Table 2:** Univariable Analyses of Progression to ESRD

	ESRD at 1 Year			ESRD During Total Follow-up		
	HR	95% CI	*P*	HR	95% CI	*P*
Age (/1 SD)	1.32	0.97-1.78	0.07	1.15	0.92-1.45	0.2
Initial SCr (/1 mg/dL)	1.26	1.19-1.33	<0.001	1.24	1.18-1.29	<0.001
% Normal glomeruli (/1 SD)	0.27	0.15-0.49	<0.001	0.34	0.23-0.49	<0.001
% Active glomeruli (/1 SD)	1.59	1.24-2.07	0.001	1.47	1.20-1.82	<0.001
% Sclerosed glomeruli (/1 SD)	1.46	1.13-1.88	0.003	1.54	1.27-1.87	<0.001
Index of chronic damage (/1 SD)	1.72	1.32-2.24	<0.001	1.78	1.42-2.22	<0.001

*Note:* SCr is treated as a continuous variable; other variables are divided into groups by 1 SD, which corresponds to 16 years for age, 33% for percentage of normal glomeruli, 30% for percentage of active glomeruli, 21% for percentage of sclerosed glomeruli, and 30% for index of chronic damage. Abbreviations: CI, confidence interval; ESRD, end-stage renal disease; HR, hazard ratio; SCr, serum creatinine; SD, standard deviation.

**Table 3:** Multivariable Analyses of Progression to ESRD

	ESRD at 1 Year			ESRD During Total Follow-up		
	HR	95% CI	*P*	HR	95% CI	*P*
SCr at presentation (mg/dL)	1.21	1.14-1.28	<0.001	1.19	1.13-1.25	<0.001
% Normal glomeruli	0.42	0.21-0.82	0.01	0.47	0.31-0.71	<0.001

*Note:* Multivariable analyses were performed using forward stepwise Cox regression. Variables used were age; index of chronic damage; percentages of normal, active, and sclerosed glomeruli (all divided by standard deviation); and SCr level at presentation (milligrams per deciliter). Abbreviations: CI, confidence interval; ESRD, end-stage renal disease; HR, hazard ratio; SCr, serum creatinine.

**Table 4:** Progression to End-Stage Renal Disease by Quartiles of Percentage of Normal Glomeruli

	Percentage of Normal Glomeruli in Quartile		Progression to End-Stage Renal Disease		
Quartile	Range	Median	%	*P*	Relative Risk (95% confidence interval)
1	0-8	0	46.6	<0.001	10.53 (4.71-23.54)
2	9-31	17	22.8	0.001	4.39 (1.86-10.40)
3	32-65	46	14.1	0.1	1.99 (0.80-5.01)
4	67-100	86.5	7.5	—	1.00 (reference)

*Note:* Global *P* < 0.001.

**Table 5:** Univariable and Multivariable Analyses of Progression to End-Stage Renal Disease in Patients Undergoing > 1 Renal Biopsy

	Univariable Analysis		Multivariable Analysis	
	Hazard Ratio (95% confidence interval)	*P*	Hazard Ratio (95% confidence interval)	*P*
Serum creatinine at biopsy 1 (/1 mg/dL)	1.16 (1.08-1.25)	<0.001		
Serum creatinine at biopsy 2 (/1 mg/dL)	1.44 (1.29-1.61)	<0.001	1.42 (1.26-1.60)	<0.001
Index of chronic damage at biopsy 1 (/1 SD)	1.35 (0.89-2.04)	0.2		
Index of chronic damage at biopsy 2 (/1 SD)	2.45 (1.73-3.61)	<0.001		
Percentage of normal glomeruli at biopsy 1 (/1 SD)	0.42 (0.26-0.69)	0.001		
Percentage of normal glomeruli at biopsy 2 (/1 SD)	0.48 (0.25-0.93)	0.03		
Rate of change in serum creatinine (mg/dL/d) (/1 SD)	0.94 (0.85-1.03)	0.2		
Rate of change in index of chronic damage (%/d) (/1 SD)	3.51 (1.92-6.42)	<0.001	2.08 (1.11-3.89)	0.02
Rate of change in percentage of normal glomeruli (%/d) (/1 SD)	1.47 (0.80-2.86)	0.2		

*Note:* All variables were used in multivariable stepwise Cox regression. Abbreviation: SD, standard deviation.

**Table 6:** Summary of Related Studies

Study	No. of Participants/Follow-up	Outcome Measures	Results^a^
Bajema et al^25^ (EC/BCR Project for ANCA assay standardization)	157/1 y	Correlations with SCr at 1 y	% normal glomeruli (−); diffuse interstitial infiltrates, tubular necrosis, % globally sclerotic glomeruli, tubular atrophy (+)
Aasarod et al^17^ (retrospective, Wagener granulomatosis only)	94/median, 42.5 mo	Correlations with SCr at 1 y	% glomeruli with crescents, % normal glomeruli (−) Multivariable analysis showed only % normal glomeruli associated with development of ESRD
Hauer et al^7^ (CYCAZAREM trial; SCr < 500 μmol/L at entry)	96/18 mo	GFR at 18 mo	39 pathologic features, GFR at entry (+); interstitial fibrosis, global glomerulosclerosis, tubular atrophy (−) In stepwise multiple regression, predictors were GFR at entry, % fibrinoid necrosis, % segmental crescents (+)
Vergunst et al^22^ (EC/BCR Project for ANCA assay standardization)	160/1 y	GFR at 1 y	16 pathologic features Stepwise regression analysis creating formula to predict GFR at 1 y including initial GFR, presence of normal glomeruli and fibrinoid necrosis, and age
Neumann et al^9^ (retrospective)	67	SCr; modified lupus nephritis activity, chronicity scoring system	At 1 y: activity index, chronicity index (+); % normal glomeruli (−) At 4 y: chronicity index (+), % normal glomeruli (−)
Hogan et al^26^ (prospective)	340/median, 49 mo		Treatment resistance associated with increased chronicity score of initial biopsy
De Lind van Wijngaarden et al^8^ (MEPEX; SCr > 500 μmol/L at entry)	100/1 y	GFR at 12 mo	Normal glomeruli (+); tubular atrophy, global glomerulosclerosis, interstitial fibrosis (−)

Abbreviations: (+), positive correlation; (−), inverse correlation; ANCA, antineutrophil cytoplasmic antibody; CYCAZAREM, cyclophosphamide versus azathioprine for remission in generalized vasculitis; EC/BCR, European Commission/Bureau Communautaire de Référence; ESRD, end-stage renal disease; GFR, glomerular filtration rate; MEPEX, methyl prednisolone or plasma exchange for severe renal vasculitis; SCr, serum creatinine.

## Box 1 Definition and Measurement of Index of Chronic Damage

**Definition of the index of chronic damage**

Percentage of renal cortex comprising atrophic areas defined as any combination of global glomerular sclerosis (but not segmental sclerosis), tubular atrophy (shrunken tubules with thickened basement membranes), interstitial fibrosis, cysts, and completely occluded vessels.

**Measurement of index of chronic damage**

A digital image is obtained of 1 routinely prepared 2-μm section of the renal biopsy stained using periodic acid–methenamine silver. Using image analysis software, the entire renal cortex (area inside the renal capsule and outside the medulla) is outlined by freehand. Cortical area is measured in arbitrary units. Areas of chronic damage are outlined by freehand, and their total area is measured and expressed as a percentage of cortical area.
